# Diagnostic Value and Effectiveness of an Artificial Neural Network in Biliary Atresia

**DOI:** 10.3389/fped.2020.00409

**Published:** 2020-08-06

**Authors:** Jia Liu, ShuYang Dai, Gong Chen, Song Sun, JingYing Jiang, Shan Zheng, YiJie Zheng, Rui Dong

**Affiliations:** ^1^Department of Pediatric Surgery, Children's Hospital of Fudan University, Shanghai Key Laboratory of Birth Defect, Shanghai, China; ^2^Department of Medicine, Pulmonary Hospital Affiliated to Tongji University, Shanghai, China

**Keywords:** biliary atresia, obstructive jaundice, diagnosis, gamma-glutamyl transpeptidase, non-invasive

## Abstract

**Objectives:** Biliary atresia (BA) is a devastating pediatric liver disease. Early diagnosis is important for timely intervention and better prognosis. Using clinical parameters for non-invasive and efficient BA diagnosis, we aimed to establish an artificial neural network (ANN).

**Methods:** A total of 2,384 obstructive jaundice patients from 2012 to 2017 and their 137 clinical parameters were screened for eligibility. A standard binary classification feed-forward ANN was employed. The network was trained and validated for accuracy. Gamma-glutamyl transpeptidase (GGT) level was used as an independent predictor and a comparison to assess the network effectiveness.

**Results:** We included 46 parameters and 1,452 patients for ANN modeling. Total bilirubin, direct bilirubin, and GGT were the most significant indicators. The network consisted of an input layer, 3 hidden layers with 12 neurons each, and an output layer. The network showed good predictive property with a high area under curve (AUC) (0.967, sensitivity 97.2% and specificity 91.0%). Five-fold cross validation showed the mean accuracy for training data of 93.2% and for validation data of 88.6%.

**Conclusions:** The high accuracy and efficiency demonstrated by the ANN model is promising in the noninvasive diagnosis of BA and could be considered as in a low-cost and independent expert diagnosis system.

## Introduction

Biliary atresia (BA), a devastating pediatric liver disease, is the major cause of liver transplantation in children (1). About 2 weeks after birth, patients start presenting clinical symptoms including progressive jaundice, acholic stool, growth retardation, and rapid serum bilirubin elevation. Cholangiography and liver biopsy pathology usually show intrahepatic and extrahepatic bile duct obstruction, hilar fiber block, liver inflammation, and fibrosis (2). Surgical intervention, mainly via Kasai portoenterostomy, is the only way to reestablish bile flow in BA (3). However, many BA patients still suffer from progressive liver fibrosis after the Kasai procedure and will eventually need liver transplantation (4).

Incidence rates of BA vary globally, with East Asia having the highest incidence of BA with a rate of about 1.7–3.7 in 10,000 live births (5, 6). While the etiology of BA is still poorly understood, timely and proper surgical intervention is widely accepted as important for management (7, 8). A Kasai procedure performed within 60 days of birth contributes to a better prognosis in BA patients (9–11). Currently, diagnosis of BA is done by surgical cholangiography and liver biopsy, both of which are invasive procedures with prolonged recovery times.

Therefore, it is important to establish an accurate and non-invasive modality for the early diagnosis of BA.

The artificial neural network (ANN) is a non-linear regression model and can be used in a computer-aided diagnosis system (12). An ANN model based on a combination of non-invasive clinical parameters from BA patients and their differential diagnosis was developed. This study aimed to evaluate its effectiveness in BA diagnosis.

## Methods

### Patient Inclusion and Data Collection

Patients with obstructive jaundice patients who were suspected of BA, admitted to the surgical department of the Children's Hospital of Fudan University, and underwent surgical cholangiography from the 2nd of January 2012 to the 30th of November 2017 were enrolled for screening. Patient information and results from laboratory tests were retrospectively obtained from the medical records. This study was approved by the Ethics Committee of the Children's Hospital of Fudan University. Informed consent was obtained from the legal guardians of all patients before enrollment in the study.

Basic information was obtained from all patients upon admission including date of birth, sex, body weight, gestational history, amalgamated malformation, and blood type. Laboratory tests including routine blood tests, urine tests, fecal tests, biochemical tests, coagulation function test, arterial blood gas analysis, TORCH and EBV screening, and hepatitis screening were performed 1–2 days before surgical cholangiography. Abdominal B ultrasound for liver, gallbladder, and spleen was also performed as imaging examination parameter.

### Data Processing

**Figure 1:**
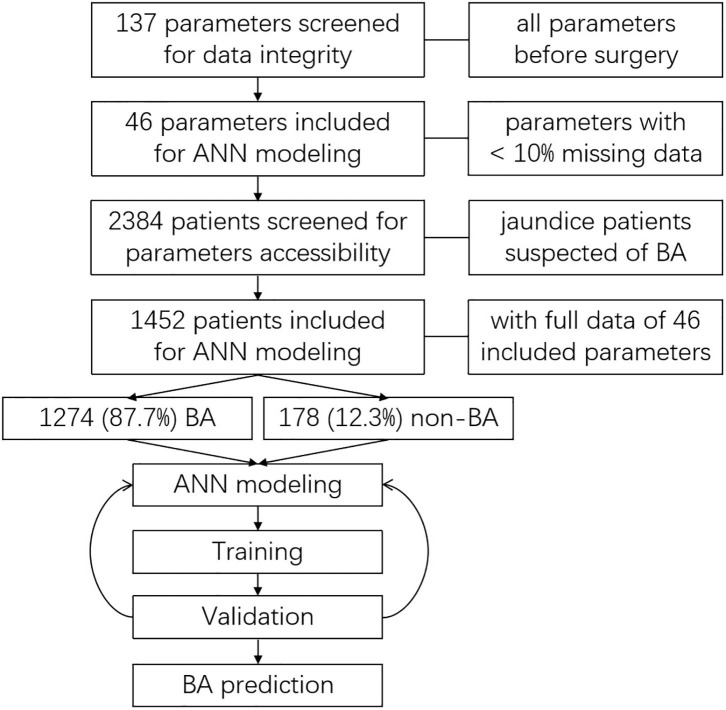
Data screening and processing for ANN modeling and training.

Altogether, 137 parameters (6 basic information parameters, 130 laboratory test parameters, and 1 imaging examination parameter, shown in Supplementary Table 1) were screened for data integrity. Parameters with a missing data ratio of <10% were included for further analyses. Afterward, clinical parameters from all enrolled patients were screened for data integrity. Only patients with full accessible data of the included parameters were included for ANN modeling (Figure 1).

Included patients were divided into two groups, the BA group and the non-BA group. Diagnosis was made by surgical cholangiography and liver biopsy pathology during surgery.

### ANN Modeling

**Figure 2:**
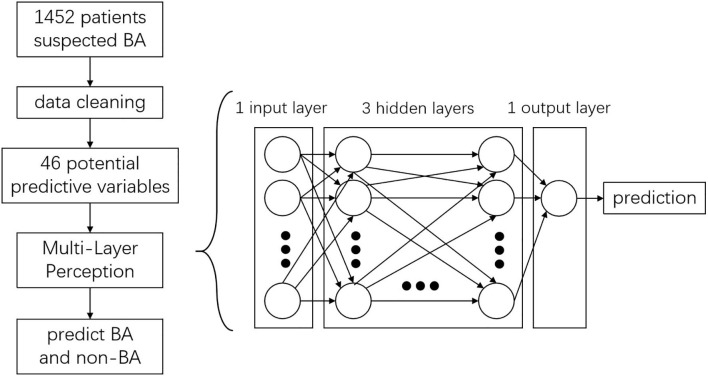
ANN modeling procedure and structure.

In this scenario, a standard binary classification problem was introduced, that is, whether the patient has BA or not. To address this, a standard binary classification feed-forward ANN model was applied. The ANN was developed using the Python programming language, with a combination of Keras and TensorFlow. The network consisted of an input layer, three hidden layers, and an output layer with one neuron (Figure 2). Each neuron contained a series of weights and biases which were multiplied, added to the inputs, and then passed through an activation function to determine what numerical value was passed from a given neuron to the next layer or output from the network. These weights and biases were optimized to obtain the best performance from the network for BA prediction.

All binary inputs were fed to the network as either 0 or 1, while continuous inputs were transformed, such that the mean of the data set was shifted to 0 and the standard deviation was normalized to 1. The initial weights on all the neurons were drawn randomly. For the hidden layer, a rectified linear unit activation function was used, while a sigmoid activation function was employed for the output layer. The Adam optimizer was used to optimize the weights and biases. Finally, a “loss function” was defined to assess the performance of the network. For this, binary cross-entropy was used, which is standard for binary classification problems. After automatic parameter tuning, the network was trained using batch sizes of 20 and optimized over 300 epochs. To limit overfitting of the network, the dropout method was employed, with 0.4 as the dropout rate for each hidden layer.

Accuracy, along with a five-fold cross validation, was used to evaluate the network. The mean values for accuracy in both training data and validation data were reported. Using ROC curves, GGT levels were used as an independent predictor to assess the predicted probability in all included subjects.

### Statistical Analysis

Continuous data were presented as means ± standard deviation (SD), and qualitative variable data were presented as percentages. The Wilcoxon rank-sum test and chi-square test were used for comparisons between the two groups as appropriate. Area under the curve (AUC), sensitivity, specificity, positive predictive value (PPV), and negative predictive value (NPV) were used to describe the predictive properties. A *P* < 0.05 was considered to be statistically significant. Statistical analysis was performed using the Python 3.6 and R software 3.5 programs.

## Results

### Patient Information and Parameter Inclusion

After data screening, 46 qualified parameters were included and analyzed. A total of 2,384 patients were screened for eligibility. Of this total, the 1,452 patients who had full data accessibility for all 46 analyzed parameters were included for further analyses and ANN modeling. Among the included patients, 1,274 (87.7%) of them were diagnosed with BA, while 178 (12.3%) were not (Figure 1). The diseases of non-BA patients included cholestasis, infant hepatitis syndrome, bile duct dysplasia, progressive familial intrahepatic cholestasis (PFIC), and Alagille syndrome. All the non-BA patients were transferred to and treated in the Internal Medicine Department.

### Parameter Analyses

The 46 included parameters were each compared between the BA and non-BA groups (shown in Supplementary Table 2). Compared to the reference values of healthy population, 10 parameters were lower in BA patients, namely, Hgb, Hct, NEUT%, TP, ALB, GLB, PA-Y, CHEW, CRES, and arterial blood pH. This indicated a deficiency in nutrition status and compromised immunity. On the other hand, eight parameters were higher in BA patients, namely, TBIL, DBIL, AST, ALP, GGT, TBA, A/G, and APTT, indicating impaired liver function and a disorder in coagulation.

There was a significantly higher proportion of female patients in the BA group than in the non-BA group. Of the other 16 clinical parameters with significant statistical difference between the two groups, five were related to routine blood function, nine were biochemical tests, one was related to coagulation function, and one was related to hepatitis. Considering the difference in mean values between both groups, its clinical significance, and its comparison to reference values in the healthy populations, the GGT level was the most significant parameter and was used as an independent predictor (BA 772.22 ± 604.41 U/L vs. non-BA 316.80 ± 380.45 U/L, *p* < 0.0001; reference value 8–57 U/L).

### ANN Modeling

**Figure 3:**
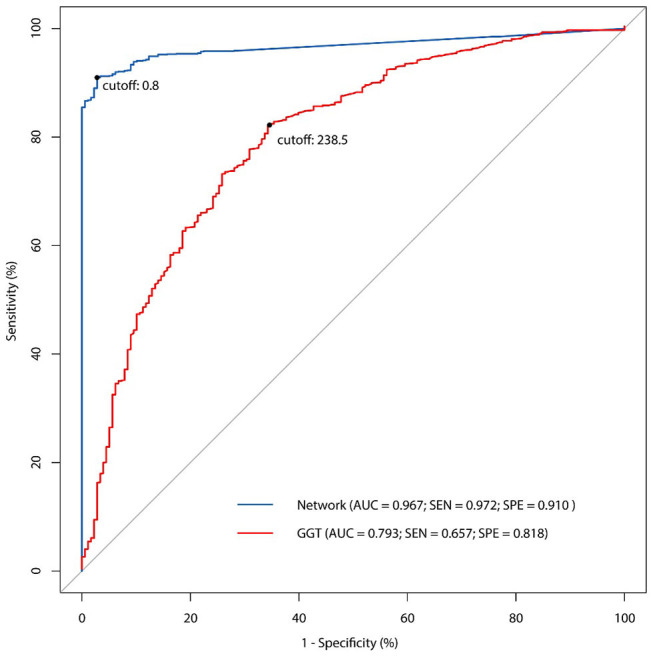
The receiver operating characteristic (ROC) curve of ANN model and comparison with single GGT predictor.

All the 46 parameters were included as variables to the input layer, the three hidden layers consisting of 12 neurons each, and the output layer consisting of one neuron. The network showed good predictive property, with a high AUC (0.967) and a cutoff point at 0.8. The sensitivity of the network was 97.2% and the specificity was 91.0%. Examining the diagnostic pattern of GGT alone, the AUC was 0.793 with a cutoff point at 238.5 U/L. The sensitivity of a single GGT predictor was 65.7% and its specificity was 81.8%. The five-fold cross validation for the ANN model revealed that the mean accuracy (95% CI) for the training data was 93.2% (92.5%−93.9%), while the mean accuracy (95% CI) for the validation data was 88.6% (88.2%−88.9%). The ROC curve (Figure 3) showed the predictive properties of both the network and GGT as a single predictor.

## Discussion

In this study, we built up an ANN model for the non-invasive diagnosis of BA, based on 46 clinical parameters from 1,274 BA patients and 178 non-BA infants. Compared to GGT as an independent predictor, this network displayed a good predictive property with a high AUC value (0.967), a sensitivity of 97.2%, and a specificity of 91.0%.

BA requires an accurate and efficient early diagnosis for timely and proper surgical intervention (7). Currently, the diagnosis of BA is based on surgical cholangiography and liver biopsy. These are invasive procedures with lengthy recovery times. As a result, the diagnostic process is prolonged and requires a significant period of post-procedural care (13). Although several diagnostic factors and prediction models have been considered for effective diagnosis of BA, they either have lacked satisfactory sensitivity and specificity or are not readily applicable or accessible in the hospital setting (14–17).

The ANN, a subfield of artificial intelligence, has shown promise in disease prediction, diagnosis, and classification. Compared to traditional diagnostic models, the ANN has the advantages of self-learning and massive data processing (18, 19). It simulates the animal nervous system with primary elements called artificial neurons (ANs). The ANs are placed in several layers; the input layer receives the variables, which are processed to subsequent hidden layers consecutively, until the output layer reaches a final prediction (20). The interactions between each layer are dependent on synaptic weights, which are determined by an iterative processing of the input variables (21). Using different mathematical methods, the synaptic weights are gradually adjusted to achieve a better predictive performance during the training and validation process (22). Therefore, the ANN can be self-generated even with small samples, and it is capable of handling a large quantity of highly complex data (20).

In our study, all clinical information and routine tests collected from the patients upon admission to our department and before surgical intervention were screened. With a quick feedback of results, all the parameters were obtained easily, resulting in a non-invasive, and efficient diagnostic model. Furthermore, sophisticated equipment is not required to obtain these parameters, which can also be measured in various hospital settings. As a result, the application of this network is easy and practical. The majority of the 46 included parameters are indicators of nutrition status and liver function, in accordance with previous findings in BA patients (6, 8). The most affected indicator in BA patients is liver function, usually presenting as increased inflammation and fibrosis. Therefore, the impaired nutrition status, compromised immunity, and coagulative dysfunction displayed are likely consequences of liver dysfunction. The differences between the reference values of healthy population and non-BA obstructive jaundice infants in our study were considered. Total bilirubin, especially direct bilirubin, and GGT were the parameters with the most significantly different values. These values are indications of a more serious obstruction of the bile duct system, as well as liver damage and dysfunction. While other affected parameters are related to nutrition, indicators of immunity and coagulation exist in both BA and non-BA jaundice patients.

GGT is an indicator for liver dysfunction. It is elevated in liver damage, especially in bile duct obstruction. GGT has been demonstrated to be a highly indicative independent predictor for BA diagnosis and prognosis (23, 24). Compared to GGT alone, our ANN model for BA diagnosis showed remarkable effectiveness, with higher sensitivity and specificity. Moreover, our network included a greater number of parameters statistically selected from multiple clinical tests, regardless of their empirical values in BA diagnosis. Since the ANN is self-promoting with the growth of data volume, and not affected by the interactions between different parameters (19), we believe our network is more objective and advanced than other linear prediction models.

However, our ANN model has some limitations. First, our variables were selected from all parameters in the medical records from the past few years and excluded large amounts of parameters with low data integrity. As a result, several potentially important factors may have been excluded prior to the ANN modeling. Therefore, due to the complex learning processes involved in the model development, it may be impossible to figure out what parameters exist in the hidden layers (the so-called black box) or to reproduce the same ANN model (12, 18). Although the dropout method was applied to avoid bias and overfitting, and our network was validated for stability, there is risk when applying this network to a larger data set from multiple institutions (25). Therefore, future validation and adjustment with integral and stable data from multiple institutions would further verify the predictive ability of this ANN model and its application in BA diagnosis.

For further applications of this ANN model, adjustment would be needed when validating and applying it in larger populations. Because of its property of self-learning and bias control, we believe that the ANN model would become more stable and accurate after synthesizing larger amount of data. Furthermore, this study provided a proof of concept that artificial intelligence could be used in BA management and individualized treatment for early detection and differential diagnosis and potentially for prognosis predictions, such as risk factor detection and survival evaluation.

In conclusion, we established an ANN model based on multiple non-invasive parameters for BA diagnosis. This network provided accurate and efficient diagnosis for BA patients. It also provides an opportunity for independent expert diagnosis for resource-limited communities in the future.

## Data Availability Statement

All datasets presented in this study are included in the article/Supplementary Material.

## Ethics Statement

The studies involving human participants were reviewed and approved by The Ethics Committee of the Children's Hospital of Fudan University. Written informed consent to participate in this study was provided by the participants' legal guardian/next of kin.

## Author Contributions

SZ, YZ, and RD: study conception and design. JL, SD, GC, SS, JJ, SZ, YZ, and RD: material preparation, data collection, and analysis. JL, SD, SS, and JJ: drafting of the manuscript. GC, SZ, YZ, and RD: critical revision. All authors: commented on previous versions of the manuscript, read, and approved the final manuscript.

## Conflict of Interest

The authors declare that the research was conducted in the absence of any commercial or financial relationships that could be construed as a potential conflict of interest.
