# Supplementary material for: Diagnostic Value and Effectiveness of an Artificial Neural Network in Biliary Atresia
Source: Front Pediatr. 2020 Aug 6;8:409. doi: 10.3389/fped.2020.00409 (PMC7438882; doi:10.3389/fped.2020.00409)
Supplement: Supplementary file 1 [file Data_Sheet_1.doc]

Supplementary Material

# Supplementary Table 1. Basic information and laboratory test parameters screened for data integrity

| Basic Information (6) | | | |
| --- | --- | --- | --- |
| age at surgery | sex^*^ | body weight | gestational history |
| amalgamated malformation | | blood type |  |
| Blood Routine (24) | | | |
| WBC^*^ | RBC^*^ | HGB^*^ | PLT^*^ |
| HCT^*^ | MCV^*^ | MCH^*^ | MCHC^*^ |
| RDW-SD^*^ | PDW^*^ | MPV^*^ | P-LCR^*^ |
| PCT | LYM%^*^ | NEUT%^*^ | MXD%^*^ |
| EO%^*^ | BASO%^*^ | LYM^*^ | NEUT |
| MXD | EO^*^ | BASO | CRP |
| Urine Test (15) | | | |
| color | transparency | WBC | RBC |
| epithelial cells | LEU | PRO | PH |
| GLU | BLD | KET | BIL |
| UBG | SG | NIT |  |
| Fecal Test (6) | | | |
| color | appearance | parasite eggs | WBC |
| RBC | phagocytes |  |  |
| Biochemical Test (34) | | | |
| TBIL^*^ | DBIL^*^ | ALT | AST^*^ |
| ALP^*^ | GGT^*^ | TBA^*^ | TP^*^ |
| ALB^*^ | GLB^*^ | A/G^*^ | PA-Y^*^ |
| TCH | TG | APOA | APOB |
| HDL-C | LDL-C | LPA | MAO |
| HBDH | RBPS | CK-MB | CHEW^*^ |
| UREA^*^ | CRES^*^ | UA | CYSC |
| K | NA | CL | CA |
| P^*^ | MG |  |  |
| Coagulation Function (7) | | | |
| PT^*^ | INR^*^ | APTT^*^ | FIB^*^ |
| TT^*^ | DDR | NFDP |  |
| Arterial Blood Gas Analysis (24) | | | |
| PH^*^ | temperature | FIO2 | pCO2 |
| pO2 | sO2 | HGB | HCT |
| NA+ | K+ | CL- | CA++ |
| GLU | LAC | H+ | SBE |
| ABE | SBC | HCO3- | CTCO2 |
| TO2 | P50E | ANION GAP | MOSM |
| TORCH and EBV screening (14) | | | |
| HSVI-IgG | HSVII-IgG | HSVI-IgM | HSVII-IgM |
| CMV-IgG | CMV-IgM | RV-IgG | RV-IgM |
| TOX-IgG | TOX-IgM | EBV-CA-IgG | EBV-CA-IgM |
| EBV-EA-IgG | EBV-NA-IgG |  |  |
| Hepatitis Screening (6) (continued) | | | |
| HBsAg^*^ | HBsAb^*^ | HBeAg^*^ | HBeAb^*^ |
| HBcAb^*^ | HCVAb |  |  |
| Imaging Examination (1) - abdominal B ultrasound for liver, gallbladder, and spleen | | | |

^*^, included parameters for ANN modeling after screening of data integrity.

Blood Routine: WBC, white blood cell; RBC, red blood cell; HGB, hemoglobin; PLT, platelet; HCT, hematocrit; MCV, mean corpuscular volume; MCH, mean corpuscular hemoglobin; MCHC, mean corpuscular hemoglobin concentration; RDW-SD, red blood cell distribution width; PDW, platelet distribution width; MPV, mean platelet volume; P-LCR, platelet-large-cell ratio; PCT, thrombocytocrit; LYM, lymphocyte; NEUT, neutrophil; MXD, monocyte; EO, eosnophils; BASO, basophils; CRP, C-reactive protein.

Urine Test: LEU, urine white blood cell; PRO, protein; GLU, glucose; BLD, urine occult blood; KET, ketone; BIL, bilirubin; UBG, urobilinogen; SG, specific graity; NIT, nitrite.

Biochemical Test: TBIL, total bilirubin; DBIL, direct bilirubin; ALT, glutamic-pyruvic transaminase; AST, glutamic oxalacetic transaminase; ALP, alkaline phosphatase; GGT, gamma-glutamyl transpeptidase; TBA, total bile acid; TP, total protein; ALB, albumin; GLB, globulin; A/G, ALB/GLB; PA-Y, prealbumin; TCH, total cholesterol; TG, triglyceride; APOA, apolipoprotein A1; APOB, apolipoprotein B; HDL-C, high-density lipoprotein; LDL-C, low-density lipoprotein; LPA, lipoprotein (a); MAO, monoamine oxidase; HBDH, hydroxybutyrate dehydrogenase; RBPS, Vitamin A alcohol binding protein; CK-MB, creatine kinase isoenzyme MB; CHEW, cholinesterase; CRES, creatinine; UA, uric acid; CYSC, cystatin C; K, potassium; NA, sodium; CL, chlorine; CA, calcium; P, phosphorus; MG, magnesium.

Coagulation Function: PT, prothrombin time; INR, international normalized ratio; APTT, activated partial thromboplastin time; FIB, fibrinogen; TT, thrombin time; DDR, D-dimer; NFDP, fibrin degradation products.

Arterial Blood Gas Analysis: FIO2, fraction of inspiration oxygen; pCO2, partial pressure of carbon dioxide; pO2, partial pressure of oxygen; sO2, oxygen saturation; LAC, lactic acid; SBE, standard base excess; ABE, actual base excess; SBC, standard bicarbonate; CTCO2, carbon dioxide content; TO2, oxygen content; P50E, partial oxygen pressure at 50% oxygen saturation; MOSM, osmotic pressure.

# Supplementary Table 2. Included parameters comparison between BA and non-BA groups

|  | Reference Value | Non-BA | BA | P-value |
| --- | --- | --- | --- | --- |
| Basic Information (1) | | | | |
| Female (%) |  | 48 (27.0) | 621 (48.7) | <0.0001^*^ |
| Male (%) |  | 130 (73.0) | 653 (51.3) |  |
| Blood Routine (19) | | | | |
| WBC (*10^9^/L) | 6-12 | 9.27 ± 3.23 | 9.45 ± 4.46 | 0.7324 |
| RBC (*10^12^/L) | 4-5.5 | 5.33 ± 26.51 | 5.53 ± 7.74 | 0.1891 |
| HGB (g/L)^a^ | 110-160 | 99.04 ± 13.38 | 99.51 ± 15.08 | 0.3336 |
| PLT (*10^9^/L) | 100-400 | 334.57 ± 149.14 | 319.78 ± 178.59 | 0.2775 |
| HCT (%)^a^ | 34-48 | 29.60 ± 3.97 | 30.25 ± 11.16 | 0.6646 |
| MCV (fL) | 73-100 | 87.51 ± 8.78 | 89.99 ± 24.45 | 0.0004^*^ |
| MCH (pg) | 26-32 | 29.19 ± 3.06 | 30.28 ± 8.91 | <0.0001^*^ |
| MCHC (g/L) | 320-380 | 333.10 ± 16.41 | 334.70 ± 21.19 | 0.0563 |
| RDW-SD (fL) | 37-54 | 52.51 ± 8.33 | 50.37 ± 6.85 | 0.0063^*^ |
| PDW (fL) | 9-17 | 12.00 ± 2.31 | 12.43 ± 3.72 | 0.1547 |
| MPV (fL) | 9-13 | 10.53 ± 1.48 | 10.79 ± 1.62 | 0.0622 |
| P-LCR (%) | 13-43 | 28.75 ± 7.70 | 29.91 ± 7.44 | 0.1064 |
| LYM% (%) | 50-70 | 59.73 ± 11.31 | 60.70 ± 18.06 | 0.2611 |
| NEUT% (%)^a^ | 30-40 | 26.57 ± 11.31 | 27.29 ± 13.37 | 0.7416 |
| MXD% (%) | 3-8 | 8.58 ± 6.89 | 7.78 ± 3.57 | 0.1065 |
| EO% (%) | 0.5-5 | 4.97 ± 3.84 | 4.20 ± 3.54 | 0.0149^*^ |
| BASO% (%) | 0-1 | 0.47 ± 0.37 | 0.39 ± 0.25 | 0.0034^*^ |
| LYM (*10^9^/L) | 4-8.4 | 5.53 ± 2.36 | 5.69 ± 3.24 | 0.4547 |
| EO (*10^9^/L) | 2.4-4.8 | 8.18 ± 103.40 | 2.60 ± 60.26 | 0.1741 |
| Biochemical Test (15) | | | | |
| TBIL (umol/L)^b^ | 3.4-17.1 | 156.47 ± 59.76 | 169.54 ± 177.55 | 0.0083^*^ |
| DBIL (umol/L)^b^ | 0-6 | 104.47 ± 39.02 | 114.20 ± 66.98 | 0.0015^*^ |
| AST (IU/L)^b^ | 15-40 | 165.30 ± 123.88 | 172.40 ± 125.61 | 0.2128 |
| ALP (IU/L)^b^ | 54-369 | 669.20 ± 303.11 | 612.90 ± 235.50 | 0.0404^*^ |
| GGT (U/L)^b^ | 8-57 | 316.80 ± 380.45 | 772.22 ± 604.41 | <0.0001^*^ |
| TBA (umol/L)^b^ | 0-10 | 136.55 ± 58.85 | 139.48 ± 67.61 | 0.6360 |
| TP (g/L)^a^ | 65-85 | 53.96 ± 7.69 | 54.61 ± 6.29 | 0.2744 |
| ALB (g/L)^a^ | 40-55 | 38.65 ± 4.86 | 38.71 ± 3.91 | 0.8907 |
| GLB (g/L)^a^ | 20-30 | 15.42 ± 4.80 | 17.03 ± 34.06 | 0.0143^*^ |
| A/G^b^ | 1.5-2.5 | 2.81 ± 1.58 | 2.59 ± 0.74 | 0.0056^*^ |
| PA-Y (mg/L)^a^ | 200-400 | 147.27 ± 75.67 | 166.47 ± 90.57 | 0.0128^*^ |
| CHEW (U/L)^a^ | 5300-11300 | 223.40 ± 113.19 | 424.60 ± 7001.06 | 0.0167^*^ |
| UREA (mmol/L) | 2.8-7.6 | 2.89 ± 1.11 | 2.77 ± 1.01 | 0.1877 |
| CRES (umol/L)^a^ | 22-90 | 16.78 ± 3.92 | 16.34 ± 6.56 | 0.0191^*^ |
| P (mmol/L) | 1.29-2.26 | 1.70 ± 0.56 | 2.06 ± 12.26 | 0.3571 |
| Coagulation Function (5) | | | | |
| PT (S) | 11-14.5 | 13.15 ± 1.59 | 13.32 ± 6.04 | 0.5452 |
| INR | 0.8-1.2 | 1.00 ± 0.16 | 1.06 ± 2.37 | 0.5163 |
| APTT (S)^b^ | 26-40 | 43.10 ± 6.73 | 46.02 ± 123.29 | 0.2771 |
| FIB (g/L) | 2-4 | 2.80 ± 0.76 | 2.63 ± 0.66 | <0.0001^*^ |
| TT (S) | 14-21 | 18.20 ± 2.09 | 18.89 ± 7.15 | 0.7018 |
| Arterial Blood Gas Analysis (1) | | | | |
| PH^a^ | 7.35-7.45 | 7.12 ± 0.44 | 7.19 ± 2.65 | 0.9044 |
| Hepatitis Screening (5) | | | | |
| HBsAg | <0.05 IU/ml | 0.06 ± 0.62 | 0.01 ± 0.04 | 0.0359^*^ |
| HBsAb (mIU/ml) | <10 | 112.34 ± 189.86 | 115.67 ± 183.92 | 0.1855 |
| HBeAg | <1 S/CO | 0.03 ± 0.07 | 0.10 ± 2.52 | 0.5226 |
| HBeAb | >1 S/CO | 0.32 ± 0.76 | 0.32 ± 0.75 | 0.7815 |
| HBcAb | <1 S/CO | 1.35 ± 1.93 | 1.29 ± 1.86 | 0.9399 |

^*^, p < 0.05.

^a^, decreased parameters in BA patients compared with the reference values of healthy population.

^b^, increased parameters in BA patients compared with the reference values of healthy population.
